# Optimizing the formation of the acquired enamel pellicle *in vitro* for proteomic analysis

**DOI:** 10.1590/1678-7757-2020-0189

**Published:** 2020-08-05

**Authors:** Vinícius Taioqui PELÁ, Talita Mendes Oliveira VENTURA, Marília Afonso Rabelo BUZALAF

**Affiliations:** 1 Universidade Federal de São Carlos Departamento de Genética e Evolução São CarlosSP Brasil Universidade Federal de São Carlos, Departamento de Genética e Evolução, São Carlos, SP, Brasil.; 2 Universidade de São Paulo Faculdade de Odontologia de Bauru Departamento de Ciências Biológicas BauruSP Brasil Universidade de São Paulo, Faculdade de Odontologia de Bauru, Departamento de Ciências Biológicas, Bauru, SP, Brasil.

**Keywords:** Pellicle, Enamel, Saliva, Proteomics, Methods

## Abstract

Saliva is the major contributor for the protein composition of the acquired enamel pellicle (AEP), a bacteria-free organic layer formed by the selective adsorption of salivary proteins on the surface of the enamel. However, the amount of proteins that can be recovered is even smaller under *in vitro* condition, due to the absence of continuous salivary flow.

**Objective:**

This study developed an *in vitro* AEP protocol for proteomics analysis using a new formation technique with different collection solutions.

**Methodology:**

432 bovine enamel specimens were prepared (4x4 mm) and divided into four groups (n=108). Unstimulated saliva was provided by nine subjects. The new AEP formation technique was based on saliva resupply by a new one every 30 min within 120 minutes at 37ºC under agitation. AEP was collected using an electrode filter paper soaked in the collection solutions according with the group: 1) 3% citric acid (CA); 2) 0.5% sodium dodecyl sulfate (SDS); 3) CA followed by SDS (CA+SDS); 4) SDS followed by CA (SDS+CA). The pellicles collected were processed for analysis through LC-ESI-MS/MS technique.

**Results:**

A total of 55 proteins were identified. The total numbers of proteins identified in each group were 40, 21, 28 and 41 for the groups CA, SDS, CA+SDS and SDS+CA, respectively. Twenty-three typical AEP proteins were identified in all groups, but *Mucin* was only found in CA and CA+SDS, while three types of PRP were not found in the SDS group. Moreover, a typical enamel protein, *Enamelin*, was identified in the CA+SDS group only.

**Conclusion:**

The new technique of the *in vitro* AEP formation through saliva replacement was essential for a higher number of the proteins identified. In addition, considering practicality, quantity and quality of identified proteins, citric acid seems to be the best solution to be used for collection of AEP proteins.

## Introduction

Saliva is formed mainly by the secretion of salivary glands. This fluid is essential for the homeostasis of the oral cavity, since it cleans, lubricates and protects the oral tissues, as well as acting as a buffering agent and source of calcium and phosphate ions for remineralization of the teeth.^1^ Moreover, saliva is the major contributor for the protein composition of the acquired enamel pellicle (AEP), a bacteria-free organic layer formed by the selective adsorption of salivary proteins on the surface of the enamel,^2^ but containing also carbohydrates, neutral lipids, phospholipids and glycolipids.^3-5^ These organic components grant important functions to the AEP that acts as a diffusion barrier, reducing the direct contact of the acids with the tooth surface, slowing down tooth dissolution.^1,6,7^

The ability of the AEP to protect the enamel surface against acids is due mainly to its protein composition, especially by the proteins present in the basal layer. These remain in the AEP after exposure to acids^8^ and are currently objects of great interest, since they might protect against dental caries and erosion. In the last few years, proteomic approaches have been used to identify these proteins^9-12^ so that they can be added to dental products which, when applied, could modify the composition of the AEP, increasing its protective potential against acids.^13^

One of the main difficulties faced in the studies involving proteomic analysis of the AEP is the small amount of proteins that can be obtained, which can impair analysis, both in *in vitro, in situ* and *in vivo*. The amount of proteins that can be recovered is even smaller under *in vitro* condition, due to the absence of continuous salivary flow. Moreover, in the *in vivo* studies available so far, AEP samples collected from 8-10 volunteers are pooled in order to obtain enough proteins to be analyzed by mass spectrometry,^10-12,14-18^ which does not allow proper assessment of the biological variation of the samples. In these *in vivo* studies, the collection of the AEP samples is done with filter paper soaked in 3% citric acid.

Recently, the proteome of the acquired pellicle formed *in situ* on ceramic specimens and collected by incubation in Tris-HCl buffer containing Triton X-100 followed by ultrasonication in RIPA buffer was analyzed from individual volunteers, with high inter-individual and inter-day consistency.^19^ However, the protocol of collection of the AEP employed by Delius 2017^19^ is not viable to be employed *in vivo*, since Triton X-100 is toxic and sonication is not possible. In addition, 0.5% dodecyl sodium sulphate (SDS) has been employed for the collection of AEP samples for analysis of individual proteins by immunoblotting,^20^ but SDS was not tested for collection of AEP samples for proteomic analyses yet.

Thus, the aim of this study was to develop an *in vitro* AEP formation protocol comparing different collection solutions for shotgun proteomic analysis. The solutions tested (3% citric acid and 0.5% SDS, alone or in combination) were chosen based on their potential to be employed under *in vivo* conditions, which would allow individual analysis and better assessment of biological variation among the volunteers in future studies.

## Methodology

### Ethical aspects and subjects

This study was approved by the local Ethics Committees (Human and Animal, protocols 86772718.0.0000.5417 and 007/2018, respectively) of Bauru School of Dentistry, University of São Paulo, SP, Brazil).

Nine young adult subjects of both genders took part in the study, after signing an informed consent document. The exclusion criteria for the volunteers were: presence of caries lesions, use of medication that could change the salivary flow, gingivitis, smoking habit, periodontitis, low salivary flow (unstimulated and stimulated flows should be greater than 0.1 and 1.0 mL/minute, respectively).

The volunteers received a kit containing a toothbrush, toothpaste and floss for oral hygiene standardization. In the morning (to avoid circadian effects),^21^ after oral hygiene (2 hours), unstimulated saliva was collected from each volunteer in tubes, kept in ice. Saliva samples were immediately centrifuged (4.500 x*g* at 4°C, 15 min). The supernatants were collected, pooled and added to a 1:100 protease inhibitor (phenylmethane sulfonyl fluoride - PMSF, N-Ethyhlmaleimide - NEM and Phenantroline).^9^ Saliva supernatants were stored at -80ºC, until use.

### Preparation of bovine specimens

Bovine incisors underwent a process of screening and cleaning (removal of soft tissue) before preparation. Each tooth was glued on an acrylic plate with thermoactive dental plaster (Kerr Corporation, Orange, CA, EUA) for the separation of the root and coronary portions. The crowns were cut using a precision cutting machine (ISOMET Low Speed Saw Buehler Ltd., Lake Bluff, IL, EUA), with two diamond discs (double-sided XL 12205 ‘high concentration’, 102 × 12.7 × 0.3 mm^3^; Extec Diamont Wafering Blade, Enfield, CT, USA) separated by a 4-mm thick spacer, in order to obtain 4 × 4 × 2 mm enamel specimens.

### Study groups

A total of 432 standardized bovine enamel specimens were obtained and divided into four groups (n=108/group), according to the solution used to collect the AEP, as follows: 1) 3% citric acid (CA)^16^; 2) 0.5% sodium lauryl sulfate (SDS)^20^; 3) CA followed by SDS (CA+SDS); 4) SDS followed by CA (SDS+CA).

### Formation of AEP *in vitro*

For the formation of the AEP, the specimens were placed in 96-well microplates in which 250 µL of saliva were added. The AEP was then allowed to form for 120 min. For the constant control of the temperature and agitation, a ThermoMixer^®^ (Eppendorf ThermoMixer^®^ C, Hamburg, Germany) was used at 37°C, under agitation. The mainly particularity in this study was the new methodology adopted regarding the resupply of saliva. For this, during the AEP formation (120 min), saliva was exchanged three times (every 30 min). This way, the previous saliva was removed and a new sample was immediately added (250 µL).

### Collection of the AEP

After the formation of AEP, the specimens were immediately withdrawn from saliva and washed with a small spray of deionized water for three seconds and air dried. The AEP was collected using an electrode filter paper 5 × 10 mm (Electrode Wick, Bio-Rad, Hercules, CA, USA) soaked in the collection solutions according with the respective group. The excess of the acid was removed with absorbent paper. For CA+SDS and SDS+CA groups, one filter paper was used for the first solution and a new filter paper was used for the second one. One filter paper was used for 6 specimens only and then resupplied by a new one.

For AEP collection, each paper soaked with their respective solution was rubbed (no pressure) on the enamel surface, with the aid of tweezers.^16^ The filter papers used to collect AEP from the specimens of the same group were placed in 2 mL tubes and stored at -80°C. The experiment was repeated for additional 2 consecutive days.

### Shotgun proteomics analysis by NanoLC-ESI-MS/MS

The methods were exactly as described elsewhere.^17^ The papers with the samples were cut into small pieces with the aid of sterile scissors and tweezers. The filter papers containing the AEP collected from 3 different days (triplicate collection) for each of the groups were pooled to obtain enough amount of AEP proteins to be submitted to the proteomic analysis.

The peptides identification was performed on a nanoACQUITY UPLC-Xevo QTof MS system (Waters, Manchester, UK). In addition, ProteinLynx Global Server (PLGS) version 3.0 was used to process and search the continuum LC-MSE data. Samples from each group were analyzed in triplicate (technical triplicates). Proteins were searched for on the *Homo sapiens* proteome database (reviewed only, UniProtKB/Swiss-Prot) downloaded on April 2017 from UniProtKB (http://www.uniprot.org/).^17^

Finally, the identified proteins were classified and assigned by biological function,^18, 22^ origin and molecular interaction (http://www.uniprot.org/) (Table S1).

**Table S1:** Classification of proteins from the acquired pellicle collected *in vitro* represented in each group

Accession number	Protein Name	Score	CA	SLS	CA + SLS	SLS + CA
P68032	Actin_ alpha cardiac muscle 1 (d, m, n, q, u, w)	65.6055	X			x
P68133	Actin_ alpha skeletal muscle (b, d, m, n, q, u, w)	65.6055	X			x
P62736	Actin_ aortic smooth muscle (b, d, m, n, q, u)	65.6055	X			x
P60709	Actin_ cytoplasmic 1 (b, m, n, q, u, w)	65.6055	X			x
P63261	Actin_ cytoplasmic 2 (a, d, g, j, n, q, u, w)	65.6055	X			x
P63267	Actin_ gamma-enteric smooth muscle (b, m, n, q, u, w)	65.6055	X			x
P04745	Alpha-amylase 1 (a, g, o, u)	452.4455	X	x	x	x
P19961	Alpha-amylase 2B (a, g, o, u)	579.3912	X	x	x	x
G5E9X6	Basic salivary proline-rich protein 1 (b, l, o, u)	155.8623	X		x	x
P02812	Basic salivary proline-rich protein 2 (b, l, o, u)	155.8623	X		x	x
Q562R1	Beta-actin-like protein 2 (b, m, n, u, w)	78.1257	X			x
Q96RL1	BRCA1-A complex subunit RAP80 (d, m, p, u)	47.3122	X			
P38398	Breast cancer type 1 susceptibility protein (b, e, m, n, p, u)	85.8217	X			
Q8N4G4	CA6 protein (a,m,t,u)	76.7328		x	x	x
P23280	Carbonic anhydrase 6 (a, g, o, u)	301.6657	X	x	x	x
Q9BXL7	Caspase recruitment domain-containing protein 11(c, e, m, n, s, w)	60.8839	X			
P08603	Complement factor H (b, m, o, u)	38.0628			x	
Q03591	Complement factor H-related protein 1 (a, m, o, w)	38.0628			x	
P01036	Cystatin-S (a, b, g, o, u)	2640.733	X	x	x	x
P09228	Cystatin-SA (a, b, g, o, u)	451.4857	x	x	x	x
P01037	Cystatin-SN (a, b, g, o, u)	2646.624	x	x	x	x
Q9UGM3	Deleted in malignant brain tumors 1 protein (f, m, n, o, v, w)	86.0094	x	x	x	x
O75928	E3 SUMO-protein ligase PIAS2 (e, m, p, u)	24.2121			x	
Q9NRM1	Enamelin (b, d, m, o, w)	15.9628			x	
P68871	Hemoglobin subunit beta (b, c, m, n, o, u, w)	293.9594		x		x
P02042	Hemoglobin subunit delta (b, c, m, n, o, u, w)	293.9594		x		x
P02100	Hemoglobin subunit epsilon (b, c, m, n, u)	293.9594		x		x
P69891	Hemoglobin subunit gamma-1(b, c, h, n, o, u, w)	293.9594		x		x
P69892	Hemoglobin subunit gamma-2 (b, c, m, n, u)	293.9594		x		x
P01876	Immunoglobulin heavy constant alpha 1 (b, e, i, j, o, u)	866.7542	x	x	x	x
P01877	Immunoglobulin heavy constant alpha 2 (b, e, i, j, o, u)	806.5165	x	x	x	x
P01591	Immunoglobulin J chain (a, b, m, o, w)	946.0537				x
Q8WYH8	Inhibitor of growth protein 5 (b, m, p, u)	95.4649			x	
Q9H1B7	Interferon regulatory factor 2-binding protein-like (b, m, p, u)	9.3029				x
P31025	Lipocalin-1 (a, b, m, o, w)	623.5907	x	x	x	x
P61626	Lysozyme C (a, b, g, i, j, o, u, w)	268.2844	x	x	x	x
Q8TAX7	Mucin-7 (b, i, k, o, u)	417.1399	x		x	
C9JTN7	Nucleolysin TIA-1 isoform p40 (b,m,n,x)	92.8821	x			
P04746	Pancreatic alpha-amylase (a, g, o, u)	1996.417	x	x	x	x
Q6S8J3	POTE ankyrin domain family member E (b, m, o, u)	65.6055	x			x
A5A3E0	POTE ankyrin domain family member F (b, m, o, u)	65.6055	x			x
P0CG38	POTE ankyrin domain family member I (b, m, o, u)	69.466				x
P0CG39	POTE ankyrin domain family member J (b, m, o, u)	69.466				x
A0A0A0MT31	Proline-rich protein 4 (b, l, p, u)	420.9096	x		x	x
P06702	Protein S100-A9 (a, b, g, i, j, n, o, q, s, u, w)	711.9667	x			x
Q9BYX7	Putative beta-actin-like protein 3 (a, m, n, q, u, w)	65.6055	x			x
Q5VSP4	Putative lipocalin 1-like protein 1 (b, m, o, x)	204.5444	x	x	x	x
P02810	Salivary acidic proline-rich phosphoprotein 1/2 (b, d, h, l, o, u, v)	420.9096	x		x	x
Q8NBW4	Sodium-coupled neutral amino acid transporter 9 (f, m, r, u, w)	255.0823	x			
Q86WA9	Sodium-independent sulfate anion transporter (c, m, s, u)	262.1345	x			
P02808	Statherin (b, e, i, l, o, u)	54090.52	x	x	x	x
P02814	Submaxillary gland androgen-regulated protein 3B (a, g, o, u, w)	3959.276	x	x	x	x
P17987	T-complex protein 1 subunit alpha (e, m, n, w)	59.5312	x			
A0A087WZY1	Uncharacterized protein (m, t, x)	420.9096	x		x	x
P25311	Zinc-alpha-2-glycoprotein (a, b, g, o, u, w)	496.7979	x			

Classification of proteins according to: General Function: a) metabolism; b) biological process; c) transport; d) structure and structural organization; e) information pathways; f) miscellanea; Function in AP: g) metabolism; h) tissue regeneration; i) antimicrobial; j) immune response; k) lubrication; l) biomineralization; m) unknown biological function; Origin: n) cytoplasm origin; o) extracellular origin; p) nucleus origin; q) cytoskeleton origin; r) intracellular origin; s) membrane origin; t) unknown protein origin; Interaction: u) protein/protein interaction; v) calcium/phosphate binding; w) other molecular interaction; x) unknown molecular interaction. The groups are: 3% citric acid (CA), 0.5% sodium lauryl sulfate (SLS), 3% citric acid plus 0.5% sodium lauryl sulfate (CA+SLS) and 0.5% Sodium lauryl sulfate plus 3% citric acid (SLS+C).

## Results

**Figure 1:**
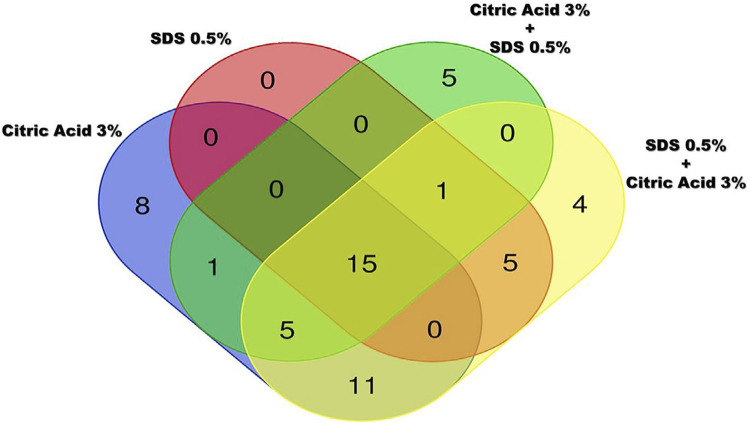
Venn Diagram with the numbers of the exclusive proteins from each group and the proteins common to two or more group

The total amount of AEP proteins recovered was very similar for all the groups, ranging between 26 and 33 µg. A total of 55 proteins were identified (Figure 1), among which are 20 proteins typically found in the AEP, such as two isoforms of Alpha-amylase, two isoforms of Basic salivary proline-rich protein, three isoforms of Cystatin, five isoforms of Hemoglobin, *Lysozyme, Mucin-7, Pancreatic alpha-amylase, Proline-rich protein 4, Protein S100-A9, Salivary acidic proline-rich phosphoprotein ½, Statherin and Submaxillary gland androgen-regulated protein 3B* (Table S1).

The total numbers of proteins identified in each group were 40, 21, 28 and 41 for CA, SDS, CA+SDS and SDS+CA, respectively. Among them, 15, 14, 14 and 9 are proteins typically found in the AEP (Table 1). Additionally, the proteins found exclusively in one of the groups was 8, 0, 5 e 4 for the groups CA, SDS, CA+SDS and SDS+CA, respectively (Table 1; Figure 1).

**Table 1:** Proteins identified in the acquired enamel pellicle formed *in vitro* on enamel specimens and collected using different solutions

Group	Accession number	Protein Name	Score
**CA**	P68032	Actin_ alpha cardiac muscle 1	65.6055
	P68133	Actin_ alpha skeletal muscle	65.6055
	P62736	Actin_ aortic smooth muscle	65.6055
	P60709	Actin_ cytoplasmic 1	65.6055
	P63261	Actin_ cytoplasmic 2	65.6055
	P63267	Actin_ gamma-enteric smooth muscle	65.6055
	P04745	**Alpha-amylase 1**	452.4455
	P19961	**Alpha-amylase 2B**	579.3912
	G5E9X6	**Basic salivary proline-rich protein 1**	155.8623
	P02812	**Basic salivary proline-rich protein 2**	155.8623
	Q562R1	Beta-actin-like protein 2	78.1257
	Q96RL1*	BRCA1-A complex subunit RAP80	47.3122
	P38398*	Breast cancer type 1 susceptibility protein	85.8217
	P23280	Carbonic anhydrase 6	144.9005
	Q9BXL7*	Caspase recruitment domain-containing protein 11	60.8839
	P01036	**Cystatin-S**	2640.733
	P09228	**Cystatin-SA**	451.4857
	P01037	**Cystatin-SN**	2646.624
	Q9UGM3	Deleted in malignant brain tumors 1 protein	86.0094
	P01876	Immunoglobulin heavy constant alpha 1	866.7542
	P01877	Immunoglobulin heavy constant alpha 2	806.5165
	P31025	Lipocalin-1	623.5907
	P61626	**Lysozyme C**	268.2844
	Q8TAX7	**Mucin-7**	417.1399
	C9JTN7*	Nucleolysin TIA-1 isoform p40	92.8821
	P04746	**Pancreatic alpha-amylase**	1996.417
	Q6S8J3	POTE ankyrin domain family member E	65.6055
	A5A3E0	POTE ankyrin domain family member F	65.6055
	A0A0A0MT31	**Proline-rich protein 4**	420.9096
	P06702	**Protein S100-A9**	711.9667
	Q9BYX7	Putative beta-actin-like protein 3	65.6055
	Q5VSP4	Putative lipocalin 1-like protein 1	204.5444
	P02810	**Salivary acidic proline-rich phosphoprotein 1/2**	420.9096
	Q8NBW4*	Sodium-coupled neutral amino acid transporter 9	255.0823
	Q86WA9*	Sodium-independent sulfate anion transporter	262.1345
	P02808	**Statherin**	54090.52
	P02814	**Submaxillary gland androgen-regulated protein 3B**	3959.276
	P17987*	T-complex protein 1 subunit alpha	59.5312
	A0A087WZY1	Uncharacterized protein	420.9096
	P25311*	Zinc-alpha-2-glycoprotein	496.7979
**SDS**	P04745	**Alpha-amylase 1**	274.6967
	P19961	**Alpha-amylase 2B**	274.6967
	Q8N4G4	CA6 protein	76.7328
	P23280	Carbonic anhydrase 6	301.6657
	P01036	**Cystatin-S**	293.6917
	P09228	**Cystatin-SA**	216.0704
	P01037	**Cystatin-SN**	274.2227
	Q9UGM3	Deleted in malignant brain tumors 1 protein	56.2783
	P68871	**Hemoglobin subunit beta**	293.9594
	P02042	**Hemoglobin subunit delta**	293.9594
	P02100	**Hemoglobin subunit epsilon**	293.9594
	P69891	**Hemoglobin subunit gamma-1**	293.9594
	P69892	**Hemoglobin subunit gamma-2**	293.9594
	P01876	**Immunoglobulin heavy constant alpha 1**	290.1472
	P01877	**Immunoglobulin heavy constant alpha 2**	9.2098
	P31025	Lipocalin-1	1070.104
	P61626	**Lysozyme C**	731.9259
	P04746	**Pancreatic alpha-amylase**	36.6661
	Q5VSP4	Putative lipocalin 1-like protein 1	1070.104
	P02808	**Statherin**	20250.94
	P02814	**Submaxillary gland androgen-regulated protein 3B**	1497.902
**CA+SDS**	P04745	**Alpha-amylase 1**	181.0646
	P19961	**Alpha-amylase 2B**	166.898
	G5E9X6	**Basic salivary proline-rich protein 1**	552.4909
	P02812	**Basic salivary proline-rich protein 2**	552.4909
	Q8N4G4	CA6 protein	67.728
	Q8N4G4	CA6 protein	47.9429
	P23280	Carbonic anhydrase 6	728.2514
	P08603*	Complement factor H	38.0628
	Q03591*	Complement factor H-related protein 1	38.0628
	P01036	**Cystatin-S**	1556.063
	P09228	**Cystatin-SA**	1013.174
	P01037	**Cystatin-SN**	205.9523
	Q9UGM3	Deleted in malignant brain tumors 1 protein	118.9028
	O75928*	E3 SUMO-protein ligase PIAS2	24.2121
	Q9NRM1*	Enamelin	15.9628
	P01876	Immunoglobulin heavy constant alpha 1	154.7424
	P01877	Immunoglobulin heavy constant alpha 2	79.6826
	Q8WYH8*	Inhibitor of growth protein 5	95.4649
	P31025	Lipocalin-1	1275.895
	P61626	**Lysozyme C**	1199.526
	Q8TAX7	**Mucin-7**	93.5897
	P04746	**Pancreatic alpha-amylase**	166.898
	A0A0A0MT31	**Proline-rich protein 4**	325.6618
	Q5VSP4	Putative lipocalin 1-like protein 1	1275.895
	P02810	**Salivary acidic proline-rich phosphoprotein 1/2**	325.6618
	P02808	**Statherin**	32088.14
	P02814	**Submaxillary gland androgen-regulated protein 3B**	1053.619
	A0A087WZY1	Uncharacterized protein	325.6618
**SDS+CA**	P68032	Actin_ alpha cardiac muscle 1	145.4612
	P68133	Actin_ alpha skeletal muscle	145.4612
	P62736	Actin_ aortic smooth muscle	145.4612
	P60709	Actin_ cytoplasmic 1	145.4612
	P63261	Actin_ cytoplasmic 2	145.4612
	P63267	Actin_ gamma-enteric smooth muscle	145.4612
	P04745	**Alpha-amylase 1**	3352.857
	P19961	**Alpha-amylase 2B**	3008.341
	G5E9X6	**Basic salivary proline-rich protein 1**	174.755
	P02812	**Basic salivary proline-rich protein 2**	174.755
	Q562R1	Beta-actin-like protein 2	72.3325
	Q8N4G4	CA6 protein	48.5939
	P23280	Carbonic anhydrase 6	836.3896
	P01036	**Cystatin-S**	1501.204
	P09228	**Cystatin-SA**	657.3894
	P01037	**Cystatin-SN**	1529.473
	Q9UGM3	Deleted in malignant brain tumors 1 protein	233.5314
	P68871	**Hemoglobin subunit beta**	761.2395
	P02042	**Hemoglobin subunit delta**	761.2395
	P02100	**Hemoglobin subunit epsilon**	761.2395
	P69891	**Hemoglobin subunit gamma-1**	761.2395
	P69892	**Hemoglobin subunit gamma-2**	761.2395
	P01876	**Immunoglobulin heavy constant alpha 1**	621.9611
	P01877	**Immunoglobulin heavy constant alpha 2**	182.5975
	P01591*	Immunoglobulin J chain	946.0537
	Q9H1B7*	Interferon regulatory factor 2-binding protein-like	9.3029
	P31025	Lipocalin-1	1312.528
	P61626	**Lysozyme C**	4389.076
	P04746	**Pancreatic alpha-amylase**	3061.443
	Q6S8J3	POTE ankyrin domain family member E	117.0785
	A5A3E0	POTE ankyrin domain family member F	117.0785
	P0CG38*	POTE ankyrin domain family member I	69.466
	P0CG39*	POTE ankyrin domain family member J	69.466
	A0A0A0MT31	**Proline-rich protein 4**	368.7032
	P06702	**Protein S100-A9**	132.0728
	Q9BYX7	Putative beta-actin-like protein 3	47.6124
	Q5VSP4	Putative lipocalin 1-like protein 1	1295.604
	P02810	**Salivary acidic proline-rich phosphoprotein 1/2**	368.7032
	P02808	**Statherin**	32670.96
	P02814	**Submaxillary gland androgen-regulated protein 3B**	1295.599
	A0A087WZY1	Uncharacterized protein	368.7032

* Proteins exclusively identified in each group. Proteins highlighted in bold are typical of the acquired enamel pellicle. The groups are: 3% citric acid (CA), 0,5% sodium dodecyl sulfate (SDS), 3% citric acid plus 0,5% sodium dodecyl sulfate (CA+SDS) and 0,5% Sodium dodecyl sulfate plus 3% citric acid (SDS+CA).

Fifteen proteins were identified in all groups (Figure 1), most of them being proteins typically described in the AEP, such as *Pancreatic alpha-amylase, Submaxillary gland androgen-regulated protein 3B, Immunoglobulin heavy constant alpha 1, Immunoglobulin heavy constant alpha 2*, two isoforms of Alpha-amylase, three isoforms of Cystatin, *Lysozyme C* and *Statherin* (Table S1).

Remarkably, *Mucin-7* was only identified in the CA and CA+SDS groups, while *Protein S100-A9* was only found in the CA and SDS+CA groups. On the other hand, isoforms of *Hemoglobin* were only detected in the SDS and SDS+CA groups. Moreover, a typical enamel protein*, Enamelin*, was identified in the CA+SDS group only. Furthermore, 3 types of PRP were not found in the SDS group (Table S1).

## Discussion

The proteomic analysis of AEP formed *in vitro* is an important tool in pre-clinical studies since it allows preliminary evaluation of preventive agents for dental caries and dental erosion. In addition, in *in vitro* studies it is possible to recover the enamel specimens over which the AEP is formed to be submitted to distinct tests, which is not feasible *in vivo*. However, to date there is only one study where the proteomic profile of the AEP formed *in vitro* was evaluated.^25^ In this sense, our main aim was to develop an *in vitro* protocol of the AEP formation using different solutions previously described in the literature to collect AEP proteins for shotgun proteomic analysis.

The main reason for such scarcity of studies is the small amount of proteins that can be recovered from the *in vitro* formed AEP, whereas that in *in vivo* condition the AEP is formed under continuous salivary flow, which is not present *in vitro*. In order to overcome this, in this study we resupplied the saliva in which the specimens were immersed every 30 min during the two-hour period of AEP formation. This procedure was successful for an *in vitro* study, since it allowed recovery of approximately 30 µg of proteins that is enough for proper proteomic analysis. In contrast, pilot studies performed for the definition of this protocol with the absence of saliva exchange demonstrated the failure in the recovery proteins of the AEP (data not shown). Despite the fact that saliva was resupplied every 30 to increase the total amount of recovered proteins, it is possible that the solution used to collect the AEP proteins may also influence the amount of recovered proteins.

To date, most of the studies available in the literature employ 3% citric acid for collected of the acquired pellicle.^9-11,14-18,23-25^ However, in these studies, the proteins collected from 8-10 volunteers are pooled in order to obtain enough amount of proteins to be analyzed by mass spectrometry, i.e., it is not possible to perform individual analysis. More recently, the pellicle proteins formed on ceramic specimens *in situ* were eluted by incubation in TRIS-HCl buffer containing SDS, followed by ultrasonication in RIPA-buffer. This procedure allowed analysis of individual samples with high inter-individual and inter-day consistency.^19^ However, it cannot be done *in vivo*, due to the necessity of sonication and to the toxicity of the detergents employed. SDS has been employed to collection AEP proteins *in vivo* in order to perform immunoblotting analysis.^20^ Since SDS is biocompatible and can be used to collected AEP proteins *in vivo*, in the present study we evaluated both 3% citric acid and 0.5% SDS, alone or in combination, in order to develop a method of collection of AEP proteins that results in large amount of proteins and can be employed in different protocols (*in vitro, in situ* and *in vivo*).

The obtained results indicate that the amount of proteins (ranging between 26 and 33 µg) recovered when these solutions were used was satisfactory, especially considering an *in vitro* study. Moreover, among the 55 proteins identified in all groups, 15 are common to all of them, most of which are classical players of the AEP. It could be expected that the combinations CA + SDS or SDS + CA could increase the total number of identified proteins, in comparison to CA or SDS only, since the acid and the detergent could be expected to remove different proteins of the AEP. However, this was not the case, since the total number of identified proteins were 40, 21, 28 and 41 for CA, SDS, CA + SDS and SDS + CA groups, respectively. It is also important to consider the quality of the identified proteins. Mucin included among the pellicle precursors^25^ and associated with lubrication^3^ and protection against erosive challenges^26^ was only identified in the CA and CA + SDS groups. This means that the use of SDS first might not remove this protein. Moreover, Enamelin, a typical enamel protein, was identified only in the CA + SDS group, indicating that this combination might remove a layer of enamel.

Thus, the results obtained indicate that the new technique develop by resupply of saliva for the AEP formation in the present study was essential for a higher number of the proteins identified by proteomics analysis. In addition, 3% citric acid is, among the tested solutions, the best one to remove AEP proteins for shotgun proteomic analysis. The amounts and quality of proteins recovered when 3% citric acid was used is satisfactory, especially considering the *in vitro* protocol of this study. Moreover, the amount of proteins recovered when CA was used (around 30 µg) might be enough to allow proteomic analysis of biological triplicates, since not assessing the biological variability is currently the major shortcoming of the proteomic studies of the AEP. It would be desirable to compare the proteomic profile of AEPs formed *in vitro, in situ* and *in vivo*, so that the results of *in vitro* and *in situ* studies can be extrapolated to the clinical condition.
